# ADVERSE CONSEQUENCES OF UNMET NEEDS AMONG INDIVIDUALS WITH DEMENTIA: THE ROLE OF PAID CAREGIVING

**DOI:** 10.1093/geroni/igz038.415

**Published:** 2019-11-08

**Authors:** Jennifer M Reckrey, Peiying Hua, Katherine Ornstein

**Affiliations:** 1 Icahn School of Medicine at Mount Sinai, New York, New York, United States; 2 Icahn School of Medicine at Mount Sinai, New York, United States

## Abstract

Individuals with dementia may enlist help from paid caregivers (i.e. home health aides, personal care attendants) to address increasingly complex care needs. Yet it is unclear what specific tasks paid caregivers help with and how this is related to the individual’s experience of unmet care needs. We used data from the 2015 National Health and Aging Trends Study (NHATS) to examine the association between type and intensity of individual paid caregiver tasks and adverse consequences of unmet needs among community-dwelling adults with dementia. Nearly one half (46%) of those with any functional impairment reported an adverse consequence of an unmet need (e.g., inability to go outside because of lack of help). Individuals who experienced adverse consequences were more likely to receive paid caregiving (37.9% vs. 21.4%, p<0.01). Those who received paid care with an individual task (e.g. toileting) were more likely to report an adverse consequence related to that task (e.g. remaining in wet or soiled clothes 50.2% vs. 41.0%, p=0.21). Paid caregivers provided a median of 15 hours of care per week. For those persons with dementia receiving less hours of care weekly, paid caregivers rarely helped with unscheduled or frequently recurring functional tasks like toileting or eating (<10% of the time). The help received by individuals with dementia was inadequate to meet their care needs. Paid caregiving will only be able to prevent adverse consequences of unmet care needs when the level of paid care provided is better matched to the care needs of the individual with dementia.

